# Prevalence, incidence, and the time trends of sleep-disordered breathing among patients with stroke: a systematic review and meta-analysis

**DOI:** 10.3389/fneur.2024.1432085

**Published:** 2024-11-18

**Authors:** Xiaofeng Su, Shanshan Liu, Cong Wang, Yan Cai, Yijing Li, Dongmin Wang, Zhaofeng Fan, Yan Jiang

**Affiliations:** ^1^Evidence-Based Nursing Center, West China Hospital, West China School of Nursing, Sichuan University, Chengdu, China; ^2^Department of Neurosurgery, West China Hospital, West China School of Nursing, Sichuan University, Chengdu, China; ^3^Department of Nursing, West China Hospital, West China School of Nursing, Sichuan University, Chengdu, China

**Keywords:** sleep-disordered breathing, stroke, epidemiology, systematic review, meta-analysis, meta-regression

## Abstract

**Background:**

Recent studies have investigated the epidemiological burden of sleep-disordered breathing (SDB) in patients with stroke; however, the results have been inconsistent, and the temporal trends of SDB after stroke remain unclear.

**Objective:**

To perform a systematic review and meta-analysis of the prevalence and incidence of post-stroke SDB, evaluate demographic and clinical characteristic predictors of post-stroke SDB, and examine temporal trends in the overall burden of post-stroke SDB.

**Methods:**

We searched PubMed, MEDLINE, Embase, Web of Science, CINAHL, and the Cochrane Library for studies reporting the burden of SDB in stroke patients published between 1 January 2010 and 30 December 2023. Two researchers independently screened the records for eligibility, extracted the data, and assessed the quality of the studies. Data were analyzed using random effects meta-analyses, and sources of heterogeneity were explored using subgroup analyses and meta-regression analyses.

**Results:**

Out of the 8,799 references retrieved, none examined the incidence of SDB after stroke. However, 85 studies from 26 countries examined the prevalence of SDB and were included. The overall prevalence of SDB, mild SDB, and moderate to severe SDB were 60.0% (95% CI, 60.0–70.0%), 30.0% (95% CI, 23.0–37.0%), and 45.0% (95% CI, 33.0–57.0%), respectively. Meta-regression revealed that sex (*p* < 0.0001) and sample size (*p* < 0.01) were sources of heterogeneity among the studies. The pooled overall prevalence of SDB remained stable over time.

**Conclusion:**

SDB is common in patients with stroke, and no reduction in the high prevalence of SDB has been observed over time, suggesting that early screening and prevention of post-stroke SDB still have not received sufficient attention. Moreover, additional studies investigating the incidence of this disease are needed to inform clinical practice.

## 1 Background

Stroke is a leading cause of death and disability worldwide (1). After a stroke, patients may experience various physical dysfunctions, including motor, speech, and swallowing dysfunctions. Although the long-term functional recovery of stroke survivors is crucial for their rehabilitation and return to normal activities (2, 3), the neuropsychiatric symptoms and complications of stroke patients have received increasing attention in recent years. Post-stroke neuropsychiatric symptoms, such as sleep-disordered breathing (SDB), chronic fatigue, and delirium, have been shown to be associated with reduced quality of life and hindered rehabilitation progress. In addition, these symptoms contribute to an increased medical burden, resulting in increased costs and an increased risk of all-cause mortality (4, 5). Among the multiple neuropsychiatric symptoms in stroke patients, SDB is one of the most common symptoms and is potentially fatal. The updated American Heart Association/American Stroke Association guidelines recommend screening for SDB among stroke patients (6). A recent study demonstrated that SDB was a significant risk factor for stroke (7). Nearly half of stroke patients experience SDB (8, 9). Poor sleep quality caused by SDB can worsen the risk of stroke onset, stroke recurrence, and poor functional recovery as well as increase the incidence of stroke-related risk factors (e.g., hypertension, atrial fibrillation, and cardiovascular disease) (10). Furthermore, SDB can adversely affect an individual’s quality of life; impair cognitive, social–emotional, or occupational functioning; and increase mortality rates, which creates a vicious cycle (11, 12).

In fact, the results of several clinical studies indicate that stroke patients have a low tolerance to hypoxemia and unstable hemodynamic changes due to concurrent SDB during hospitalization, especially in the acute phase. Continuous positive airway pressure therapy, which is the gold standard treatment for SDB, should be initiated as soon as possible (13, 14). However, assessing the burden of SDB in individuals with stroke is challenging. For example, stroke patients with impaired consciousness may struggle to cooperate, whereas those with severe physical dysfunction may have limitations during polysomnography (PSG) monitoring, such as the inability to move both upper limbs, which affects pulse oxygen monitoring. Moreover, a controlled environment for sleep monitoring is required and variations in individual tolerance may affect the assessment of results (15). Katzan et al. found that stroke patients often reported physical dysfunction and limited social engagement but rarely reported SDB-related symptoms, suggesting that the true impact of SDB in stroke patients may be masked, posing a challenge to timely risk assessment and the implementation of tailored interventions (16).

Many studies have reported the impact of SDB on stroke or the role of regular sleep in stroke recovery (17–19). A previous meta-analysis published in 2019 searched databases from inception to April 7, 2017, and included 89 studies investigating the prevalence of SDB after stroke and transient ischemic attack (20). However, our initial literature search revealed that more than 40 relevant articles were published afterward, and the reported burden of SDB varied considerably across studies. Furthermore, no systematic reviews have been published to assess the time trends of SDB burden in stroke patients. Therefore, the primary objective of this systematic review and meta-analysis was to examine the overall prevalence and incidence of SDB in stroke patients. The secondary objectives were to examine the prevalence and incidence of different severities of SDB, changes in overall SDB prevalence and incidence over time, and differences in the prevalence and incidence of SDB by gender, region, publication year, phase of stroke, study design, and SDB assessment methods.

## 2 Methods

### 2.1 Study design and registration

This systematic review and meta-analysis was conducted in accordance with the Preferred Reporting Items for Systematic Reviews and Meta-Analyses (PRISMA) 2020 statement (21) and the Meta-analysis of Observational Studies in Epidemiology (MOOSE) reporting guidelines (22). The protocol was registered in the international prospective register of systematic reviews (PROSPERO registration number CRD42023443328).

### 2.2 Search strategy and selection criteria

Two authors independently and systematically searched the PubMed, MEDLINE, Embase, Web of Science, CINAHL, and Cochrane Library databases to identify cross-sectional, longitudinal studies that reported the prevalence of SDB or different severities of SDB in individuals who experienced hemorrhagic stroke, ischemic stroke, or transient ischemic attack. Furthermore, we searched the reference lists of key reviews and meta-analyses. An initial scoping search was performed using PubMed to collate relevant keywords and medical subject headings. The initial combination of search terms was as follows: (“stroke” OR “cerebrovascular disorders” OR “brain infarction” OR “brain ischemia” OR “cerebrovascular accident*” OR “ischemic stroke” OR “hemorrhagic stroke” OR “ischemic attack, transient”) AND (“sleep disordered breathing” OR “sleep apnea syndromes” OR “sleep apnea, obstructive” OR “sleep apnea, central”) AND (“prevalence” OR “epidemiology” OR “incidence”). Next, we modified the search strategy to suit each database. Only human studies published from 1 January 2010 to 30 December 2023 were included. Our complete search strategy is available in Supplementary Table 1.

The inclusion and exclusion criteria were established according to the Population, Intervention, Comparators, Outcomes, and Study (PICOS) framework, as shown in Table 1. In addition, if multiple studies used overlapping data, we selected the study with the largest sample size. SDB was diagnosed based on the International Classification of Sleep Disorders, Diagnostic and Statistical Manual of Mental Disorders, or International Classification of Diseases (23) and assessed by PSG or questionnaires.

**Table 1:** Inclusion and exclusion criteria for the systematic review.

	Inclusion	Exclusion
Participants	Patients with stroke (no restrictions on age).	Individuals free of stroke.
Exposure	SDB (using PSG).	Non-SDB/Pre-existing comorbid SDB.
Comparators	Not applicable.	Not applicable.
Study outcome	Prevalence and/or incidence of severity mild to severe SDB in individuals after hemorrhagic stroke, ischemic stroke, or TIA. Prevalence of SDB according to stages after stroke: acute (<1 month), subacute (1–3 month), and chronic (>3 month) phase.	Comparing baseline characteristics between two groups (with or without SDB)/An intervention on two baseline groups (with or without SDB).
Study design	Observational study (Cross-sectional study and prospective cohort study or retrospective cohort study of longitudinal study). For longitudinal studies: individuals with stroke continuously tracking over time to observe SDB incidence. For cohort studies: individuals with exposure or not exposed to specific factors and clearly defined as stroke, follow up new-onset SDB after stroke.	Experimental studies (randomized control/nonrandomized control) studies/Qualitative studies/reviews.
Study period and language	Published between January 2010 and October 2023. Published in English.	Studies published prior to 2010. Non-English language articles.
Type of publication	Only peer-reviewed full-text papers.	Editorials/conference abstracts/duplicate (multiple) publication/posters.

### 2.3 Study selection

We imported the results from each database into the reference management software package EndNote and removed duplicates. When the results of the same study were reported in multiple publications, we included only the article reporting the largest sample size in the data synthesis. Next, two authors reviewed the titles and abstracts of the retrieved studies and excluded studies that were clearly not relevant (e.g., studies focusing on patients with cardiovascular disease, animal studies, or studies that examined periodic limb movements during sleep as the outcome) by using the Rayyan systematic review application (24) and screened independently the remaining references for relevance based on the full eligibility criteria. Any disagreements were resolved by discussion or by consulting a third reviewer (YJ).

### 2.4 Data extraction

The data were extracted by two independent investigators (XFS and SSL) using a standardized data extraction sheet. The data were then cross-checked for agreement, and any disagreements were resolved by consulting a third investigator (YJ). The following data were extracted: first author, publication year, country, study design, different stroke subtypes, phase of stroke, mean/median age, percentage of males, source of patients, tools used for SDB assessment, sample size, etc. If any data were unclear or unavailable, attempts were made to contact the study authors.

### 2.5 Risk of bias assessment

Two reviewers independently assessed the quality of each survey by using the Joanna Briggs Institute’s Critical Appraisal Checklist for Prevalence Studies (25), and any discrepancies were resolved by consensus. The Joanna Briggs Institute’s Critical Appraisal Checklist for Observational Studies consists of 9 items across 3 domains, namely, participants (questions 1, 2, 4, and 9), outcome measurements (questions 6 and 7), and statistics (questions 3, 5, and 8). This tool is used to evaluate the overall quality of prevalence studies. Two authors independently conducted the appraisal (XFS and SSL), and disagreements were resolved through discussion or by a third reviewer (YJ) where required.

### 2.6 Data synthesis

All the statistical analyses were conducted with Stata (version 16.0) software. The primary outcome in this systematic review and meta-analysis was the prevalence of SDB in stroke patients. The 95% confidence intervals (95% CIs) were calculated. Prevalence estimates were calculated by pooling study-specific estimates using the DerSimonian and Laird random effects model (26). The heterogeneity among the studies was assessed using Cochran’s Q statistic. The magnitude of heterogeneity was measured using the I-square (*I^2^*) statistic. The *I^2^* statistic ranged from 0 to 100% (an *I*^2^ of 0 to 25% indicated no or mild heterogeneity, 25–50% indicated moderate heterogeneity, and *I*^2^ > 50% indicated substantial heterogeneity) (27). If significant heterogeneity was not observed, the fixed effects model was used to calculate the pooled prevalence and 95% CI of SDB; otherwise, a random effects model was used.

Subgroup analyses and meta-regressions were performed to explore potential sources of heterogeneity. Subgroup analyses were performed based on the following study characteristics: study country, proportion of male patients, study design, phase of stroke, tools used for SDB assessment, and study design. We investigated publication bias by constructing funnel plots and assessing the significance of Egger’s weighted regression test. The trim-and-fill method, which was developed by Duval and Tweedie (28), was used to adjust the pooled effect sizes for publication bias. This method estimates the number of missing studies. Meta-analysis was only performed when an outcome was reported by at least three original studies. The threshold for statistical significance was set at *p* < 0.05, and all tests were two-sided.

## 3 Results

### 3.1 Search results and study selection

**Figure 1:**
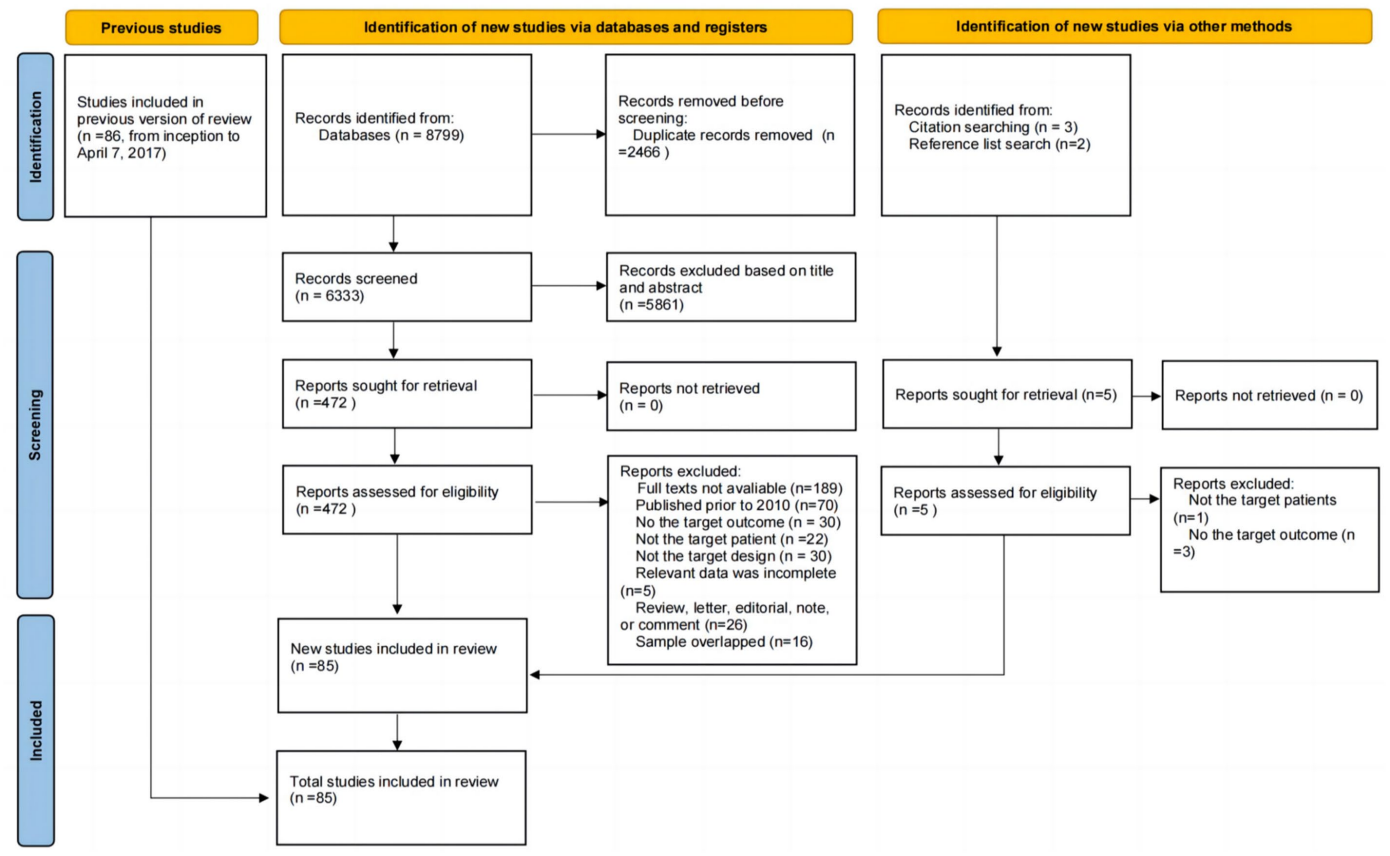
PRISMA flowchart.

The search strategy yielded a total of 8,799 unique citations, and duplicates were removed (*n* = 2,466). After screening the titles, 5,429 studies were excluded. After screening the abstracts, 432 studies were excluded. Thus, 472 articles remained for full-text evaluation. Of those studies, the number and reason for exclusion were as follows: Thirty studies did not report the target outcome, the full text of 189 studies was unavailable, 22 studies did not include the target patient group, 30 studies had an inappropriate study design, 70 studies were published prior to 2010, 16 studies had overlapping samples, and 5 studies had incomplete data. Furthermore, five potentially eligible studies were identified by searching the reference lists of relevant studies; however, only one of these studies fully met the eligibility criteria, while the other four studies were excluded because they did not report prevalence data or did not examine the target patient group. Ultimately, a total of 85 studies were included in this updated systematic review and meta-analysis, consisting of 61 new studies (29–89) and 24 older studies (11, 90–112). The details of the selection process are shown in Figure 1.

### 3.2 Study characteristics

The characteristics of the 85 included studies are shown in Table 2. The included studies were published between 2010 and 2023, with the largest number (*n* = 12) published in 2023. More than half of the included studies were published in 2017 or later. Among the 88 included observational studies, 74 were cross-sectional studies (including 69 single-center and five multicenter studies), and 11 were baseline data or at a certain time point based on longitudinal studies (including nine single-center and two multi-center study). Altogether, these studies included 5,714,316 patients with stroke, including those with ischemic stroke, transient ischemic attack, and hemorrhagic stroke. The sample sizes across the 85 reviewed studies ranged from 23 to 5,690,773.

**Table 2:** Characteristics of the included studies.

First author (year)	Country	Study design	Sample size	Age (years), mean (SD)/median (IQR)	Male%	Subtype of stroke	Phases of stroke	Tools of SDB assessment	SDB (%)
Zhu et al., 2023	China	Based on prospective cohort	180	18–85	67.8	Noncardiogenic stroke	Acute phase	⑥	72.8
Tayade et al., 2023	India	Cross-sectional study	103	≥18 mean 50.7 (11.7)	73.8	Ischemic stroke	Subacute/chronic phase	⑤	33
Plomaritis et al., 2023	Greece	Based on prospective cohort	126	mean 60.3 (10.9)	67.5	Ischemic stroke	Acute phase	⑥	78.6
Lisabeth et al., 2023	USA	Longitudinal study	414	≥18 mean 63.96 (10.93)	56.5	Ischemic stroke	Acute phase	⑥	35
Bochkarev et al., 2023	Russia	Cross-sectional study	281	18–89 mean 67	52	Ischemic stroke	Acute phase	⑥	64.8
Patel et al., 2023	USA	Multi-center, cross-sectional study	5,690,773	NR	47.7	Ischemic stroke	Acute phase	⑩	3.3
Lin et al., 2023	China	Cross-sectional study	103	≥18, median 63 IQR 59–63	71.8	Ischemic stroke	Acute phase	⑥	90.3
Korostovtseva et al., 2023	Russia	Based on prospective cohort	328	18–80, mean 65.7 (13.6)	55.2	Ischemic stroke	Acute phase	⑥	67.1
Hong et al., 2023	Korea	Cross-sectional study	250	>19,mean 63.1 (13.5)	72.8	Ischemic stroke	Acute phase	⑤	70.4
Hoang-Anh et al., 2023	Vietnam	Cross-sectional study	56	mean 67.70 (11.07)	53.6	Ischemic stroke	Subacute phase	⑥	71.4
Duss et al., 2023	Switzerland	Multi-center, longitudinal study	437	18–85, mean 65 (13.0)	63.6	Ischemic stroke, TIA	Acute phase	③	33
Brunetti et al., 2023	Italy	Cross-sectional study	174	≥18, mean 67.3 (11.6)	54.6	Ischemic stroke	Acute phase	⑥	51.1
Liu et al., 2023	China	Cross-sectional study	283	≥18, mean 65 (12.0)	64	TIA/stroke	Acute phase	④	60.1
Zhu et al., 2022	China	Cross-sectional study	94	18–75	70.2	Ischemic stroke	Acute phase	⑥	62.8
Zhang et al., 2022	China	Cross-sectional study	1,354	≥18, mean 61.58 (10.71)	78	Hemorrhagic/ischemic stroke, and TIA	Acute phase	①	99.2
Springer et al., 2022	USA	Multi-center, cross-sectional study	1,312	≥18, median 64 IQR 57–74	53	all types of stroke	Acute/subacute phase	⑥	98.6
Simonsen et al., 2022	Denmark	Longitudinal study	99	>18, median 68 IQR 36–88	55.6	Ischemic stroke	Acute phase	⑥	56.6
Schütz et al., 2022	USA	Multi-center, cross-sectional study	1,215	≥45	NR	all types of stroke	Chronic phase	④	61.1
Rafi et al., 2022	India	Longitudinal study	67	≥18	59.7	Ischaemic/haemorrhagic storke	Subacute phase	⑥	77.6
Huhtakangas et al., 2022	Finland	Based on prospective cohort	204	≥18	NR	Ischemic stroke	Acute phase	②	91.2
Griesbach et al., 2022	USA	Cross-sectional study	103	NR	NR	Ischaemic/haemorrhagic storke	Chronic phase	⑥	76.7
Edrissi et al., 2022	USA	Cross-sectional study	5,469	NR	48.7	Ischemic stroke	Acute phase	⑥	3.1
Baillieul et al., 2022	France	Based on prospective cohort	185	NR	NR	Stroke or TIA	Chronic phase	⑥	NR
Šiarnik et al., 2021	Slovakia	Cross-sectional study	120	mean 64.0 (12.2)	NR	Ischemic stroke	Acute phase	⑥	NR
Riglietti et al., 2021	Switzerland	Cross-sectional study	60	18–75 mean 60.8 (9.6)	0	Ischemic stroke	Acute phase	②	68.3
Gottlieb et al., 2021	Australia	Cross-sectional study	82	≥18 mean 69.61 (7.4)	0	Ischemic stroke	Chronic phase	⑥	NR
Folgueira et al., 2021	Spain	Cross-sectional study	53	≥18 mean 67 (12)	62.3	Ischemic stroke	Acute phase	⑥	NR
Estai et al., 2021	Australia	Cross-sectional study	39	≥18 mean 72.3 (10)	71.8	All types of stroke	Acute phase	⑥	97.4
Domínguez-Mayoral et al., 2021	Spain	Cross-sectional study	72	≥18 mean70.46 (10.83)	69.4	Ischemic stroke	Acute phase	⑥	84.7
Chen et al., 2021	China	Cross-sectional study	109	mean 59	83.5	Ischemic stroke	Acute phase	⑥	80.7
Petrie et al., 2021	USA	Cross-sectional study	68	NR	NR	hemorrhagic stroke, TIA	Acute phase	⑥	79.4
Yoon et al., 2020	Korea	Cross-sectional study	305	NR	NR	Ischemic stroke	Acute phase	⑥	83.3
Slim et al., 2020	Canada	Cross-sectional study	102	median 9 IQR 6–14	55.9	Ischemic stroke	Chronic phase	⑧	25.5
Pajediene et al., 2020	Sweden	Cross-sectional study	66	18–75 mean 60.3 (10.6)	66.7	Ischaemic/haemorrhagic storke	Acute phase	⑥	18.2
Ott et al., 2020	Switzerland	Cross-sectional study	166	35–75	72.3	all types of stroke	Acute phase	⑥	80.1
McKee et al., 2020	USA	Cross-sectional study	224	≥18	90.6	Ischemic stroke	Subacute phase	⑥	53.6
Kisabay Ak et al., 2020	Turkey	Cross-sectional study	60	18–55 median 44.5 IQR 34–51	38.3	all types of stroke	Aacute phase	⑥	71.7
Huhtakangas et al., 2020	Finland	Cross-sectional study	204	≥18 mean67.7 (13.4)	62.7	Ischemic stroke	NR	②	80.9
Haula et al., 2020	Finland	Cross-sectional study	95	≥18	55.8	Ischemic stroke or TIA	Aacute phase	⑥	63.2
Castello-Branco et al., 2020	Brazil	Cross-sectional study	99	≥18 mean 57.5 (13.2)	60.6	Ischaemic/haemorrhagic storke	Aacute phase	⑤	NR
Brown et al., 2020	USA	Cross-sectional study	1,330	≥45	53	Ischemic stroke	Chronic phase	④	67
Nair et al., 2019	India	Cross-sectional study	102	mean 71.5	68.6	Ischemic stroke	Aacute phase	⑥	30.4
Matsuura et al., 2019	Japan	Cross-sectional study	433	mean 66.5	62.6	Ischemic stroke	Subacute phase	⑥	87.3
Li et al., 2019	China	Cross-sectional study	86	≥18 mean60.3 (12.1)	76.7	Ischemic stroke	Aacute phase	⑥	77.9
Brown et al., 2019	USA	Cross-sectional study	842	median 65 IQR 57–76	53.1	Ischemic stroke	Aacute phase	④	63.1
Zhang et al., 2018	China	Multi-center, based on prospective cohort	183	≥18 mean 63.44 (10.94)	55.7	All types of stroke	NR	⑥	61.2
Yaddanapudi et al., 2018	USA	Cross-sectional study	115	≥18 mean 64 (12)	0	Ischemic stroke	Subacute phase	⑥	65.2
Tazartukova et al., 2018	Russia	Cross-sectional study	56	NR	NR	All types of stroke	Aacute phase	⑥	67.9
Losurdo et al., 2018	Italy	Cross-sectional study	140	≥18 mean 66.9 (11.9)	54.3	Ischemic stroke	Aacute phase	②	51.4
Lisabeth et al., 2018	USA	Cross-sectional study	298	≥18, median 68	NR	Hemorrhagic stroke	Chronic phase	②	47
Festic et al., 2018	USA	Cross-sectional study	989	median 75 IQR 64–84	47.8	Ischemic stroke	Aacute phase	⑥	19.2
Slonkova et al., 2017	Czech Republi	Cross-sectional study	68	≥18	76.5	All types of stroke	Aacute phase	⑥	61.8
Scherbakov et al., 2017	Germany	Cross-sectional study	101	35–89 mean 69 (12)	61.4	Ischemic stroke	Aacute phase	⑨	57.4
Sarfo et al., 2017	Ghana	Cross-sectional study	200	>16 median 62 IQR 52–72	52.5	All types of stroke	Chronic phase	①	49.5
Ryan et al., 2017	Canada	Cross-sectional study	23	≥18 mean 66.4 (13.7)	47.8	All types of stroke	Aacute phase	⑥	78.3
Ponsaing et al., 2017	Denmark	Cross-sectional study	63	≥18 median 64 IQR 57–74	63.5	stroke/TIA	Aacute phase	⑥	NR
Menon et al., 2017	India	Based on prospective cohort	99	≥18 mean 60.1 (14)	67.7	Ischemic stroke	Aacute phase	⑥	60.6
Lisabeth et al., 2017	USA	Cross-sectional study	549	median 65 IQR 57–76	55	Ischemic stroke	Aacute phase	④	68.5
Kumar et al., 2017	India	Cross-sectional study	50	20–85 mean 54.66 (12.4)	62	All types of stroke	Aacute phase	⑥	78
Kim et al., 2017	Korea	Cross-sectional study	241	≥18 mean 64.2 (11.9)	60.6	Ischemic stroke, TIA	Aacute phase	⑦	19.9
Huhtakangas et al., 2017	Finland	Cross-sectional study	246	≥18	65.9	Ischemic stroke	Aacute phase	②	75.6
Fisse et al., 2017	Germany	Cross-sectional study	142	NR	65.5	Ischemic stroke	Aacute phase	⑥	60.6
Tur et al., 2016	Spain	Cross-sectional study	97	mean 61 (13)	76.3	Ischemic stroke	Aacute phase	③	74.2
Lutohin et al., 2016	Russia	Cross-sectional study	54	≥18 median 66I QR 57–72	59.3	Ischemic stroke	Aacute phase	②	92.6
Koo et al., 2016	USA	Cross-sectional study	164	≥18 mean 62 (11.3)	64	Ischemic stroke, TIA	Aacute phase	⑥	81.1
Ifergane et al., 2016	Israel	Cross-sectional study	43	≥18	30.2	All types of stroke	Aacute phase	⑥	86
Boulos et al., 2016	Canada	Cross-sectional study	69	≥18 mean 68.3 (14.2)	47.8	stroke/TIA	Subacute phase	⑥	46.4
Stahl et al., 2015	USA	Cross-sectional study	73	≥18 mean 59.5 (11.6)	78.1	Ischemic stroke	Aacute phase	⑥	79.5
Chen et al., 2015	China	Cross-sectional study	127	>16 median 61.3 IQR 53.6–72.7	72.4	Ischemic stroke	Subacute phase	⑥	NR
Väyrynen et al., 2014	Finland	Cross-sectional study	42	NR	NR	Ischemic stroke, TIA	Aacute phase	⑥	57.1
Shibazaki et al., 2014	Japan	Cross-sectional study	97	≥18 mean 68.1	56.7	Hemorrhagic stroke	Aacute phase	⑥	93.8
Ramos et al., 2014	USA	Cross-sectional study	176	mean 60 (12)	52.8	Ischemic stroke	Aacute phase	①	44.3
Lefèvre-Dognin et al., 2014	France	Cross-sectional study	45	mean 60.9 (11.5)	66.7	All types of stroke	Subacute phase	⑥	62.2
Kepplinger et al., 2014	Germany	Cross-sectional study	61	18–75 mean 64 (8)	47.5	Ischemic stroke	Aacute phase	⑥	91.8
Shibazaki et al., 2014	Japan	Cross-sectional study	150	NR	NR	Ischemic stroke, TIA	Aacute phase	⑥	84
Ciccone et al., 2014	Italy	Multi-center, cross-sectional study	335	≥18 mean 64	67.5	Ischemic stroke, TIA	Aacute phase	②	60.3
Cereda et al., 2014	Switzerland	Multi-center, cross-sectional study	37	≥18	81.1	Ischemic stroke, TIA	Subacute phase	⑥	NR
Ahn et al., 2014	Korea	Cross-sectional study	293	40–90 mean 68.4 (10.5)	54.3	Ischemic stroke	Aacute phase	⑥	63.1
Xu et al., 2014	China	Cross-sectional study	59	≥18 mean 59.98 (11.01)	52.5	Ischemic stroke	Aacute phase	⑥	55.9
Hsieh et al., 2014	China	Cross-sectional study	71	45–90 mean 67.1 (10.8)	66.2	Ischemic stroke	Aacute phase	⑥	NR
Camilo et al., 2014	Brazil	Cross-sectional study	66	≥18 mean 57.6 (11.5)	81.8	Ischaemic/haemorrhagic storke	Aacute phase	⑥	78.8
Aaronson et al., 2014	Netherlands	Cross-sectional study	56	NR	62.5	All types of stroke	Subacute phase	⑥	46.4
Chen et al., 2014	Bosnia and Herzegovina	Cross-sectional study	200	NR	NR	Ischaemic/haemorrhagic storke	Aacute phase	①	78
Chan et al., 2014	Canada	Cross-sectional study	66	≥18	72.7	stroke/TIA	Aacute phase	⑥	62.1
Brooks et al., 2014	Canada	Cross-sectional study	45	≥18 mean 67 (18)	62.2	Ischaemic/haemorrhagic storke	Subacute phase	⑥	91.1

SDB, sleep-disordered breathing; TIA, transient ischemic attack; ①, berlin questionnaire; ②, cardiorespiratory polygraphy; ③, respiratory polygraphy; ④, home sleep apnoea testing (HSAT); ⑤, STOP-BANG questionnaire; ⑥, polysomnography (PSG); ⑦, sleep Obstructive apnea score optimized for Stroke (SOS); ⑧, pediatric sleep questionnaire (PSQ); ⑨, transthoracic impedance records; ⑩, International Classification of Diseases; NR, not reported.

**Figure 2:**
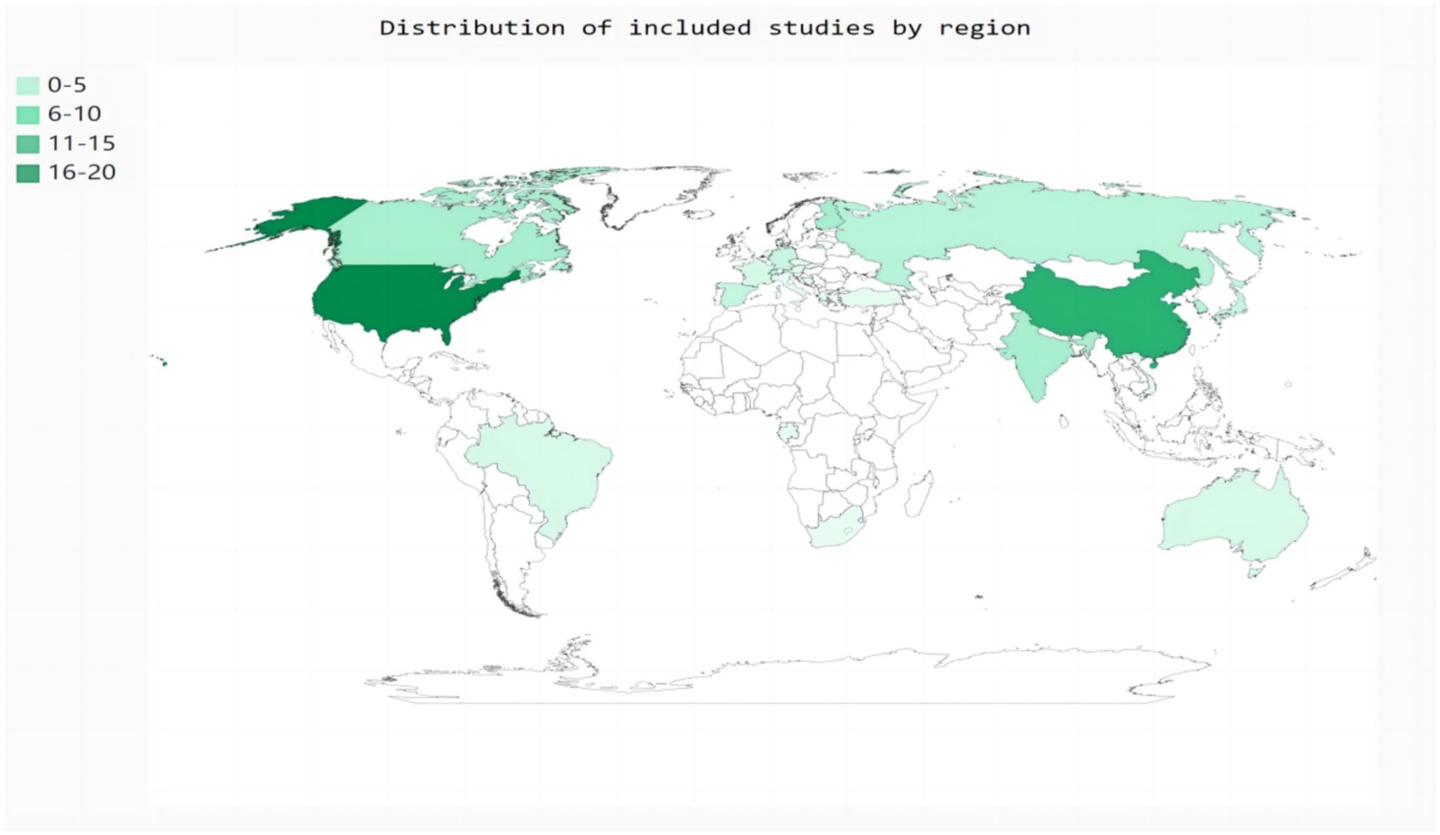
Distribution of included studies by region.

Eighty-two studies included hospital-based inpatients and outpatients (including stroke units, neural psychiatric disorders and mental health centers, and tertiary care centers). Five studies included population-based samples: three in the United States, one in Canada, and one in China. However, four studies did not report the study setting. The studies were conducted across 26 countries (Figure 2). The diagnostic tools used for SDB varied among studies. Most studies (*n* = 59) used PSG to assess SDB; eight studies used cardiorespiratory polygraphy; five studies used home sleep apnea testing; four studies used the Berlin questionnaire; three studies used the STOP-Bang score; two studies used respiratory polygraphy; one study used the Sleep Obstructive Apnea Score optimized for Stroke; one study used transthoracic impedance records; one study used the Pediatric Sleep Questionnaire; and one study used the International Classification of Diseases, Ninth Revision, Clinical Modification. Seventy-six of the 85 studies reported the total prevalence of SDB, twenty-three studies reported the severity of SDB, and no studies reported the incidence of SDB. Moreover, more than half of the studies (*n* = 48) were designed to explore the prevalence of SDB in stroke patients with a range of chronic diseases rather than in patients with stroke alone. The stroke phase was reported in 83 out of the 85 studies. Strokes were identified using MRI (*n* = 9), CT (*n* = 5), or MRI or CT (*n* = 64); some studies lacked a detailed account of the stroke diagnosis method (*n* = 7). Forty-four studies focused on ischemic stroke only; forty studies included patients with ischemic stroke, transient ischemic attack, or hemorrhagic stroke; and one study used another stroke classification. Fifteen studies did not report the sex of the patients. Twelve studies did not report the ages of the patients. Twenty-five studies reported the median age, age range(s), or minimum age of stroke patients. The mean age across the remaining 48 studies ranged from 50.7 to 71.5 years.

### 3.3 Methodological quality of the included studies

**Figure 3:**
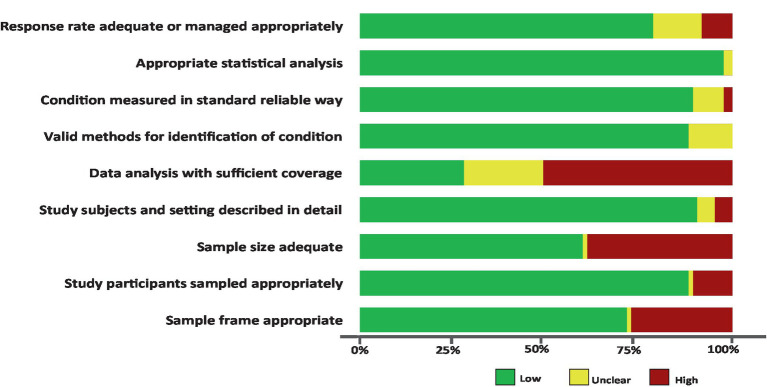
Risk of bias assessment summary table across all studies. *No weights were applied for different studies.

The quality rating assessment results are presented in the Supplementary Table 2. Across each domain in all studies, more than 76.0% of the studies had a low risk of bias, more than 7.0% exhibited a moderate risk of bias, and more than 15% showed a high risk of bias. Specifically, only <30% of the studies met the criterion of having sufficient coverage in the data analysis. The other domains, including response rate adequacy, appropriate statistical analysis, standard measurements, detailed description of the study subjects and setting, appropriate sampling of study participants, and valid methods for identifying conditions, were found to be adequate for assessing the prevalence of SDB in more than 80% of the studies. Additionally, the remaining items, such as appropriate sample size and sample frame, were adequate for more than 60% of the studies for addressing the target population (Figure 3).

### 3.4 Prevalence of SDB

**Figure 4:**
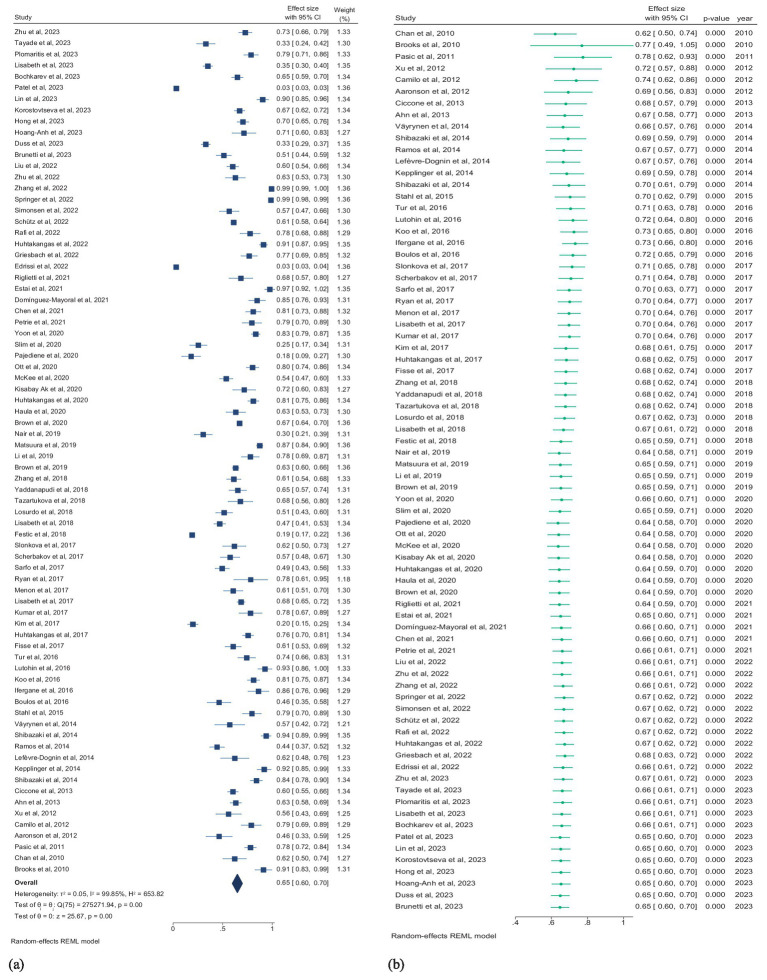
(a) Forest plot of overall prevalence of SDB in patients with stroke. (b) Forest plot of cumulative prevalence of SDB in stroke patients by year. CI, confidence interval.

Seventy-six studies (5,713,479 patients) assessed the prevalence of SDB, and studies reporting the prevalence of only the subtypes of SDB were excluded from this pooled analysis. According to the random effects model, the overall pooled prevalence of SDB in patients with stroke was 65.0% (95% CI, 60.0–70.0%; *I^2^* = 99.85) (Figure 4a). The pooled prevalence of mild and moderate-to-severe SDB were 30.0% (95% CI, 23.0–37.0%; *I^2^* = 94.87) and 45.0% (95% CI, 33.0–57.0%; *I^2^* = 94.86), respectively (Supplementary Figure 1).

### 3.5 Temporal trends in SDB prevalence

Multiple mergers were conducted in chronological order to dynamically assess the cumulative impact of publication year on the primary result. A cumulative time trend analysis of publication years revealed that the overall prevalence of SDB in stroke patients remained relatively stable from 2010 to 2023, fluctuating between 64 and 78%. The effect size also ranged from 0.49 to 1.05 over this period (*P*_for trend_ < 0.01; Figure 4b).

### 3.6 Subgroup analyses

**Figure 5:**
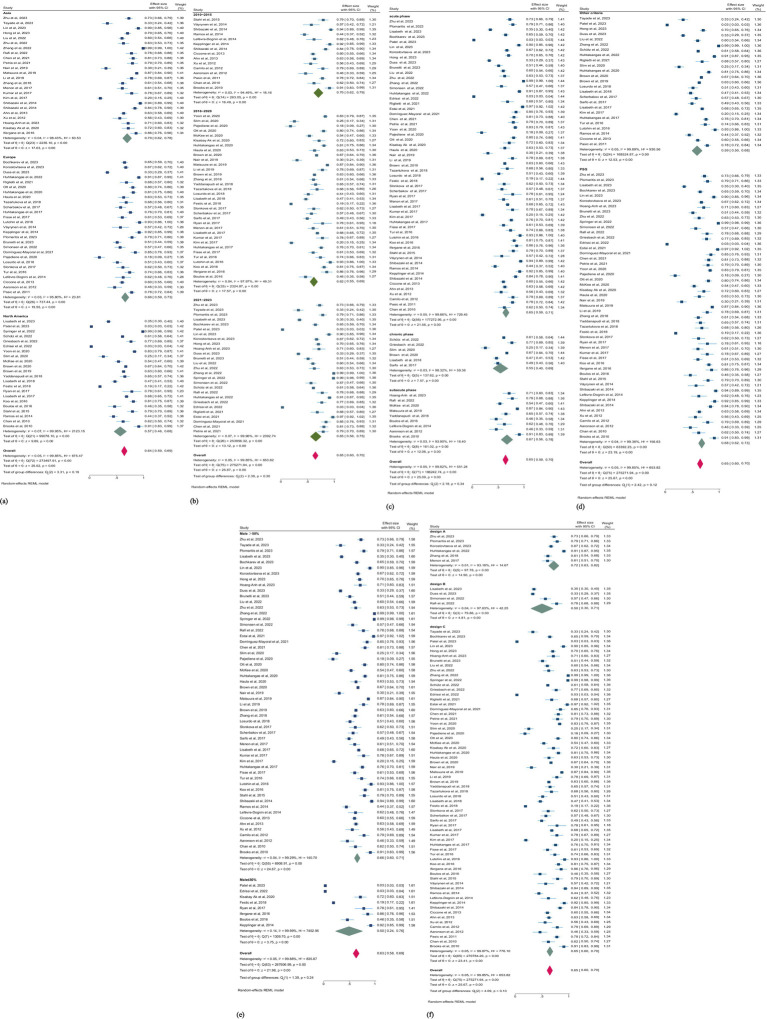
(a) Forest plot of prevalence of SDB in patients with stroke by region. (b) Forest plot of prevalence of SDB in patients with stroke by publication year. CI, confidence interval. (c) Forest plot of prevalence of SDB in patients with stroke by phases of stroke. (d) Forest plot of prevalence of SDB in patients with stroke by the tools of SDB assessment. CI, confidence interval. (e) Forest plot of prevalence of SDB in patients with stroke by gender. (f) Forest plot of prevalence of SDB in patients with stroke by study design. CI, confidence interval. Design A: Based on prospective cohort. Design B: longitudinal study. Design C: Cross-sectional study.

We conducted subgroup analyses to explore the discrepancy of the primary outcome regardless of the observed heterogeneity (Table 3). Our analysis across regions, study years, phases of stroke, gender, study designs, and assessment methods. Overall, studies in North America, Asia, and Europe exhibit geographical variances in prevalence. Among 22 studies conducted in North America (5,704,419 patients), the prevalence of SDB is the lowest (57.0%; 95% CI, 46.0–68.0%). Conversely, among 24 studies in Asia (4,563 patients), the prevalence is the highest (70.0%; 95% CI, 62.0–78.0%). The prevalence in 26 studies in Europe (3,955 patients) lies between North America and Asia. From a temporal perspective, the prevalence of SDB from 2010 to 2015 was 70.0% (95% CI, 62.0–79.0), from 2016 to 2020 was 62.0% (95% CI, 55.0–69.0), and from 2021 to 2023 was 65.0% (95%CI, 56.0–75.0). This indicates that the prevalence of SDB post-stroke fluctuates across different time periods, yet there is no evident reversal in the prevalence risk. In various phases of stroke, the prevalence of SDB also demonstrates differences, although the heterogeneity after stratification is not significantly reduced. In the chronic stage, the prevalence of SDB was the lowest. In contrast, the prevalence of SDB was highest in the subacute stage (Figures 5a–c), suggesting the alterations in SDB risk during different recovery periods post-stroke. Additionally, we performed stratified analyses according to gender (male ≤50% and male >50%), study design (cross-sectional study, cross-sectional analysis based on longitudinal study, and cross-sectional analysis based on prospective cohort), and SDB assessment method (PSG and other criteria). While some differences are present, these factors do not significantly affect the overall prevalence of SDB post-stroke (all *p* > 0.05; Figures 5d–f). This implies that in addition to the aforementioned possible interfering factors, the potential influencing factors of the prevalence of SDB after stroke remain a crucial issue worthy of attention.

**Table 3:** Summary of meta-analysis results.

Variable	Number of studies	Sample size	Patients with SDB	Effect model	Pooled estimates (%)	95% CI	Heterogeneity	
							*I* ^2^	*p*
Prevalence of SDB								
Overall	76	5,713,479	196,809	Random	65.00%	0.60–0.70%	99.85	<0.001
Mild	14	2,853	869	Random	30.0%	23.0–37.0%	94.87	<0.001
Moderate–severe	9	1,297	655	Random	45.0%	33.0–57.0%	94.86	<0.001
Region								
North America	22	5,704,419	190,386	Random	57.00%	46.0–68.0%	99.95	<0.001
Asia	24	4,563	3,490	Random	70.00%	62.0–78.0%	98.43	<0.001
Europe	27	3,955	2,544	Random	66.00%	59.0–73.0%	95.8	<0.001
Publication year								
2010 ~ 2015	15	1,764	1,197	Random	70.00%	62.0–79.0%	94.49	<0.001
2016 ~ 2020	24	7,942	4,674	Random	62.00%	55.0–69.0%	97.97	<0.001
2021 ~ 2023	27	5,703,773	190,938	Random	65.00%	56.0–75.0%	99.85	<0.001
Phases of stroke								
Acute phase	57	5,711,632	195,163	Random	65.00%	59.0–71.0%	99.85	<0.001
Subacute phase	6	19,222	9,006	Random	67.00%	56.0–78.0%	93.9	<0.001
Chronic phase	9	13,184	4,771	Random	55.00%	40.0–69.0%	98.32	<0.001
SDB assessment								
PSG	51	13,685	5,671	Random	65.00%	62.0–73.0%	99.36	<0.001
Other criteria	25	5,713,188	196,596	Random	59.00%	50.0–65.0%	99.89	<0.001
Gender								
Male ≤ 50%	8	5,740,057	207,038	Random	50.00%	24.0–78.0%	99.99	<0.001
Male > 50%	56	13,176	9,196	Random	66.00%	60.0–71.0%	99.29	<0.001
Study design								
Design A	6	2,137	1,205	Random	72.00%	63.0–82.0%	93.18	<0.001
Design B	4	1,345	617	Random	50.00%	30.0–71.0%	97.63	<0.001
Design C	66	5,711,342	195,604	Random	65.05	60.0–70.0%	99.87	<0.001

SDB, sleep-disordered breathing; Design A: based on prospective cohort. Design B: longitudinal study. Design C: cross-sectional study.

### 3.7 Meta-regression

According to the multivariate meta-regression model, the proportion of males (aOR = 1.54, 95% CI = 1.43–1.67; *p* < 0.0001) and the sample size (aOR = 0.99, 95% CI = 0.99–1.01; *p* < 0.01) were related to the prevalence of SDB, accounting for 64.31 and 10.24% of the variance in SDB prevalence, respectively (Supplementary Table 3). The study region, phase of stroke, region, and SDB assessment methods were not significantly associated with the prevalence of SDB (all *p >* 0.05).

### 3.8 Publication bias

**Figure 6:**
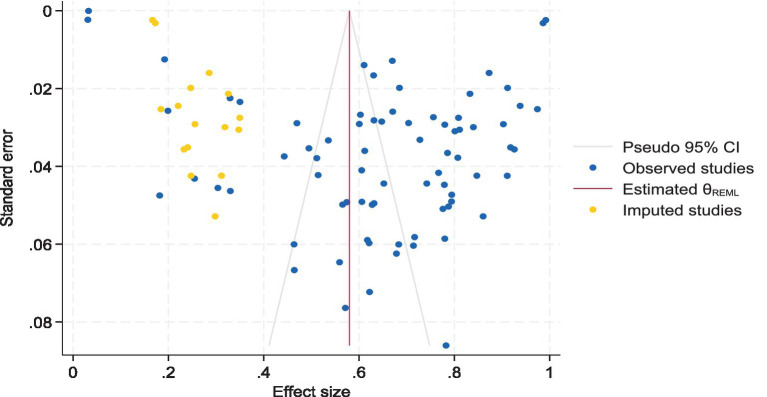
Funnel plot for the pooled prevalence of SDB after the trim and fill method.

We assessed publication bias using Egger test. The results indicated evidence of bias regarding the prevalence of SDB (*t* = 7.72; 95% CI = 0.023–0.040; *p* < 0.05). Nevertheless, based on our trim and fill analysis, we contend that this bias was minimal. Consequently, the pooled prevalence of SDB ranged from 64.7% (95% CI 59.8–69.7%) to 58.0% (95% CI 52.9–63.1%, Figure 6).

## 4 Discussion

### 4.1 Principal findings

Our meta-analysis of 85 studies published between 2010 and 2023 across 26 countries revealed that SDB burden was common among stroke patients. The overall prevalence of SDB, the prevalence of mild SDB, and the prevalence of moderate-to-severe SDB were 65, 30, and 45%, respectively. SDB most commonly occurs in the acute phase of stroke, and the overall prevalence of SDB is highest in Asia. The temporal trends of overall SDB prevalence were stable.

According to the time trend analysis conducted herein, the prevalence of SDB in stroke patients fluctuated between 62 and 77% from 2010 to 2023. Overall, the trend of the prevalence of SDB in stroke patients was stable. Nevertheless, enhancing screening for SDB in stroke patients and improving health education related to SDB in both stroke patients and their caregivers are particularly important. This includes enhancing sleep health literacy and health management methods among stroke patients and their caregivers, in turn enhancing the comprehensive health management of nonfunctional disorders in stroke to mitigate the impact of negative feedback regulation (i.e., SDB-stroke-SDB) on the risk of stroke recurrence. Moreover, the stable prevalence of SDB over time might suggest that existing interventions are to some extent effective. However, it also implies the need for new or improved methods to further reduce its prevalence. For instance, continuous positive airway pressure (CPAP), the gold standard intervention for SDB, has not decreased the prevalence of SDB after stroke. This indicates that potential factors such as the coverage, continuity, or patient compliance of CPAP intervention still need to be further considered. Additionally, the stable trend of the prevalence of SDB after stroke over time highlights the necessity to strengthen public education and the training of medical personnel to enhance the recognition of SDB after stroke. From the specific time period of the trend change in the prevalence of SDB, it can be observed that special public health events or economic fluctuations caused by events might be potential factors influencing the screening and intervention effects of SDB.

Several recent observational studies, including both prospective and retrospective cohort studies, have assessed the prevalence of SDB in stroke patients, but the results of these studies have been inconsistent (67, 73, 80, 82, 85, 88). Our systematic review and meta-analysis provided the latest pooled estimates of the prevalence of SDB in patients with stroke, and the findings were generally consistent with those of prior reports (20). The slight difference in prevalence findings may be attributed to disparities in sample sources and sizes, regional variations, cultural contexts, SDB assessment methods, and the timing of evaluations across studies. Moreover, SDB is a common disease, and its diagnosis relies primarily on PSG. Frequent awakenings during sleep is not only a symptom reported by stroke patients but also a characteristic manifestation of SDB. With respect to the pathological changes associated with stroke, worsening cerebral ischemia and hypoxia ultimately cause irreversible damage to neurons and the release of excessive excitatory amino acids and other toxic substances to disrupt the sleep–wake mechanism, which further increase the risk of SDB (9). In addition, physical dysfunction after stroke can lead to changes in nocturnal sleep posture, and an increase in supine sleep is one of the main causes of SDB (15). Thus, physicians at stroke centers recommend performing polysomnography for stroke patients to detect early signs of SDB. The potential risk of SDB in stroke patients without typical clinical manifestations is often overlooked in the stroke unit. In our meta-analysis, more than 50% of the studies included participants who had experienced a stroke and had an average age exceeding 60 years, which revealed atypical clinical manifestations of SDB in elderly individuals.

Taken together, our pooled results suggest that SDB burden is commonly observed in stroke patients, which serves as a reminder for medical professionals to carefully identify the underlying signs of SDB in stroke patients, particularly at night. Moreover, early clinical screening is necessary for stroke risk diagnosis and generalized treatment during the acute phase and healthcare during the recovery phase, as well as for preventing the exacerbation of SDB caused by conventional sleep aid medications.

### 4.2 Subgroup analysis and meta-regression findings

The analyses revealed that the prevalence of SDB after stroke was highest in Asia, followed by Europe and North America. Regional differences in the prevalence of SDB may be attributed to the limited ability to evaluate SDB diagnoses, which rely on a single sleep study or other diagnostic criteria. This approach may not adequately capture the actual sleep status of patients. Additionally, discrepancies in the sleep environment (e.g., hospital sleep centers or home monitoring) could introduce potential bias into the results. These findings indirectly indicate varying levels of emphasis on managing SDB in patients with stroke across different regions. Moreover, it is noteworthy that the majority of the research discussed in this meta-analysis originates from Asia, followed by Europe and North America. Limited research has been conducted in Oceania and North America, and related research in Africa is lacking. It is not feasible to perform subgroup analysis based on race and continent. Available epidemiological research on the prevalence of SDB in stroke patients is limited on some continents. Insufficient funding, limited regional focus, and a shortage of well-trained professionals are significant factors that impact the advancement and continuity of research. Specifically, some regions in Asia may have relatively insufficient investment in routine screening, diagnosis, and treatment of SDB. For example, the treatment costs related to SDB have not been fully included in medical insurance reimbursement, which may affect that some potential cases are not detected at early stage or patients’ initiative for diagnosis is not high due to costs, leading the aggravation of post-stroke SDB and thus being detected in large numbers. Secondly, the gold standard for post-stroke SDB is PSG, but the judgment of abnormal patterns often combines instrument types and manual analyze of professional sleep technician. In addition, some lifestyle and genetic factors in Asia may also be related to the relatively high prevalence of post-stroke SDB. Finally, cultural and socioeconomic factors, such as work pressure, the availability of social support systems, and differences in patients’ awareness of health issues may also cause differences in the prevalence of post-stroke SDB between different regions.

Subgroup analysis based on sex revealed that the prevalence of SDB was higher in studies with a proportion of males >50% than in studies with a proportion of males <50%. This result was similar to those of previous findings (68). The influence of gender on the prevalence of SDB after stroke may include physiological structure differences, hormone levels and sleep patterns. In addition, different living habits related to gender differences may interfere with the heterogeneity between studies (68). There is greater deposition of fat in the upper respiratory tract and abdomen of males, and males have longer airways than females; these differences may contribute to the increased vulnerability to airway collapse among males (113). Moreover, the clinical manifestations of SDB in females differ from those in males and are impacted by age-related and physiological states, such as menopause and pregnancy. Furthermore, compared with males, females commonly report atypical symptoms, including daytime fatigue, low energy intake, insomnia, morning headaches, mood disturbances, and nightmares (68, 114). Due to these differences in “atypical” clinical presentation, females with SDB are often underdiagnosed and undertreated compared with males. Therefore, healthcare professionals should focus on the potential signaling symptoms of SDB in female stroke patients and conduct timely screening and assessment.

In addition, the prevalence of SDB was highest between 2010 and 2015, followed by a slow decrease from 2016 to 2020. However, a slight increase occurred from 2021 to 2023. Research has indicated that SDB associated with stroke is connected to neurobiology, social psychology, and other contributing factors (115). Thus, additional research is needed to determine whether this increase is related to SDB resulting from anxiety and depression induced during the COVID-19 pandemic. For other outcomes, the heterogeneity between the subgroup analyses did not decrease, suggesting that the overall prevalence of SDB was not associated with the study design (116). Based on the use of SDB assessment tools, we found that PSG was more effective than other assessment methods for screening for the prevalence of SDB in stroke patients. In addition, the prevalence of SDB was greater among individuals with acute stroke than among individuals with stroke in the subacute and chronic phases. This difference could be attributed to the acute manifestations of stroke, including oropharyngeal dysfunction and hypoglossal nerve dysfunction, which contribute to retroglottic collapse and increase the risk of airway collapse.

The overall heterogeneity among studies was high, and the intergroup heterogeneity did not decrease following subgroup analysis. The *I^2^* values exceeded 90% across groups. Previous systematic reviews of prevalence studies reported similar findings (20). However, our meta-regression analysis further revealed that sex and sample size accounted for the high heterogeneity in the overall prevalence estimates. Different sample sizes may become potential sources of heterogeneity by affecting statistical power and so on. Boulos et al. also reviewed on the possible influence of sample size in SDB (116).

### 4.3 Strengths and limitations

Our study had some limitations. First, after searching multiple databases, no direct studies on the incidence of SDB after stroke could still be found. Therefore, a comprehensive analysis of its incidence could not be conducted. However, our study points out the direction for future research. Second, there was still substantial heterogeneity among the included studies. However, heterogeneity is a inevitable problem for meta-analyses of observational studies (117, 118). Moreover, we conducted a meta-analysis of studies that met the inclusion criteria to examine the prevalence of SDB in stroke patients. We analyzed the sources and extent of study heterogeneity through subgroup analyses and multivariable random effects meta-regression analyses. Nevertheless, subgroup analysis revealed significant heterogeneity in terms of prevalence. Although we used a random effects model to deal with heterogeneity, potential factors such as methodological differences among different studies, sample size, population characteristics, model of measurement tools, measurement methods, environment, region, and uncontrollable factors (such as differences in hypoxia tolerance) still have an impact on the results. For example, there may be biases in the diagnostic criteria or patient selection in the included studies, which may overestimate or underestimate the true prevalence of SDB. Future studies can adopt more unified diagnostic criteria, increase sample diversity, and implement more stringent research designs to reduce heterogeneity and improve the reliability of research results. The meta-regression findings indicated that sex plays a significant role in the heterogeneity. However, due to the lack of data on the pooled number of SDB cases stratified by sex in most studies, further analyses could not be conducted. Third, due to certain studies provide information only on the median age or the minimum age threshold of the overall population included, thereby precluding the possibility of conducting meta-regression models to elucidate the impact of mean age on the prevalence of SDB among stroke patients. Finally, there is a lack of available data regarding the prevalence of SDB based on the location of the stroke. The thalamus, hypothalamus, basal ganglia, brainstem reticular structure, and base of the frontal lobe are implicated in the regulation of sleep, and stroke that occurs in these anatomical regions may lead to the development of SDB (115). Comparing variations in prevalence across different locations of stroke is important for the development of risk management policies. Hence, future research should also investigate the incidence of SDB in various stroke locations.

### 4.4 Policy, clinical and research implications

Our review identified several aspects that could be addressed in future research. First, our updated pooled estimates indicate a high prevalence of SDB in stroke patients, underscoring the importance of early assessment for warning signs of SDB in this population. There is currently a gold standard method for diagnosing SDB. However, the prevalence of SDB remains high, which means that it has failed to garner sufficient clinical attention. A small proportion of stroke patients undergo regular sleep monitoring, while the majority of these patients only seek treatment at sleep centers when they experience severe SDB. Hence, it is often difficult to determine the exact timing at which patients experience SDB based on observational studies. Second, in the routine clinical diagnosis and treatment of stroke and nursing care, complications related to SDB (i.e., daytime sleepiness, intermittent irregular snoring, and frequent nocturnal awakenings) are often overlooked in favor of focusing on long-term functional recovery (i.e., motor dysfunction or cognitive impairment) in stroke survivors. The aforementioned scenario complicates the assessment of the influence of concurrent SDB on long-term prognosis and functional recovery after stroke. Routine sleep screening of stroke patients is crucial for comprehensive health management and accurate care of physical and mental symptoms. In addition, the findings emphasize the need for healthcare professionals to establish routine screening programs to proactively evaluate the prevalence of SDB in stroke patients and to promptly implement early interventions and accurate care. Optimize screening and intervention measures for SDB after stroke, including but not limited to: integrating the SDB screening process in stroke units, using portable PSG monitoring equipment for early diagnosis, and conducting multidisciplinary team cooperation to ensure that patients receive timely and appropriate treatment. Moreover, the inclusion of sleep monitoring could be considered in routine health screenings for stroke survivors in primary care within the community or as an integral component of the care offered to patients experiencing acute or subacute stroke in inpatient care facilities. Given that not all stroke patients experience overt SDB, it is imperative for future research to investigate the protective factors that may mitigate SDB after stroke. At the same time, on the basis of strengthening the training of medical staff and raising public awareness of SDB, rationally optimize the allocation of resources to ensure that necessary SDB screening and management services can also be obtained in regions with limited resources. Furthermore, in different regions, especially in regions with a high prevalence of SDB, screening and management strategies should be formulated according to local medical resources, cultural and economic conditions. Finally, the disparities in the prevalence of SDB based on sex, region, and phase of stroke require attention, as they may affect the accurate prediction of the burden of SDB after stroke. Overall, this study offers valuable insights for the formulation and implementation of preventive measures and healthcare strategies.

## 5 Conclusion

SDB burden is common among stroke patients. Our latest pooled estimate of the overall prevalence of SDB in stroke patients helps to raise awareness among healthcare personnel regarding the evaluation of early warning signs or “atypical” symptoms of SDB after stroke, especially those who have the closest contact with patients in clinical care. According to our analysis, it is necessary to improve the management of SDB after stroke. Individuals in the acute stage of stroke and male stroke patients are considered high-risk populations, whereas it is challenging to assess SDB among female stroke patients.
