# Supplementary material for: Prevalence, incidence, and the time trends of sleep-disordered breathing among patients with stroke: a systematic review and meta-analysis
Source: Front Neurol. 2024 Nov 18;15:1432085. doi: 10.3389/fneur.2024.1432085 (PMC11609221; doi:10.3389/fneur.2024.1432085)
Supplement: Supplementary file 1 [file Data_Sheet_1.docx]

# Supplementary material

| **Table of contents** |  |
| --- | --- |
| eTable 1: The searching strategies for each database. | Page 2 to page 5 |
| eTable 2. Risk of bias in included studies (Joanna-Briggs Institute’s critical appraisal checklist). | Page 6 to page 8 |
| eTable 3: Results of multivariate meta-regression analysis of SDB | Page 9 |
| eFigure 1. Forest plot of prevalence of mild SDB and moderate-severe SDB among stroke patients | Page 9 |

**eTable 1.** The search strategies.

| **Database** | **Search Strategies** |
| --- | --- |
| Pubmed | #1 Search: "Sleep Apnea Syndromes"[MeSH Terms] OR "sleep apnea, obstructive"[MeSH Terms] OR "sleep apnea, central"[MeSH Terms] OR "Obesity Hypoventilation Syndrome"[MeSH Terms]  #2 Search: "apnea syndrome sleep"[Title/Abstract] OR "Sleep Apnea Syndrome"[Title/Abstract] OR "Sleep Hypopnea"[Title/Abstract] OR "hypopnea sleep"[Title/Abstract] OR "hypopneas sleep"[Title/Abstract] OR "Sleep Hypopneas"[Title/Abstract] OR "apnea sleep"[Title/Abstract] OR "apneas sleep"[Title/Abstract] OR "Sleep Apnea"[Title/Abstract] OR "Sleep Apneas"[Title/Abstract] OR "Mixed Central and Obstructive Sleep Apnea"[Title/Abstract] OR "sleep apnea mixed"[Title/Abstract] OR "Mixed Sleep Apnea"[Title/Abstract] OR "Mixed Sleep Apneas"[Title/Abstract] OR "Hypersomnia with Periodic Respiration"[Title/Abstract] OR "sleep disordered breathing"[Title/Abstract] OR "breathing sleep disordered"[Title/Abstract] OR "sleep disordered breathing"[Title/Abstract] OR "apneas obstructive sleep"[Title/Abstract] OR "Obstructive Sleep Apneas"[Title/Abstract] OR "sleep apneas obstructive"[Title/Abstract] OR "Obstructive Sleep Apnea Syndrome"[Title/Abstract] OR "Obstructive Sleep Apnea"[Title/Abstract] OR "OSAHS"[Title/Abstract] OR "syndrome sleep apnea obstructive"[Title/Abstract] OR "sleep apnea syndrome obstructive"[Title/Abstract] OR "apnea obstructive sleep"[Title/Abstract] OR "Sleep Apnea Hypopnea Syndrome"[Title/Abstract] OR "syndrome obstructive sleep apnea"[Title/Abstract] OR "Upper Airway Resistance Sleep Apnea Syndrome"[Title/Abstract]  #3 Search:"stroke"[MeSH Terms] OR "Cerebrovascular Disorders"[MeSH Terms] OR "Brain Infarction"[MeSH Terms] OR "Brain ischemia"[MeSH Terms] OR "Ischemic Stroke"[MeSH Terms] OR "ischemic attack, transient"[MeSH Terms]  #4 Search: "cerebrovascular accident*"[Title/Abstract] OR "cva cerebrovascular accident"[Title/Abstract] OR "Cerebrovascular Apoplexy"[Title/Abstract] OR "apoplexy cerebrovascular"[Title/Abstract] OR "vascular accident brain"[Title/Abstract] OR "brain vascular accident*"[Title/Abstract] OR "cerebrovascular stroke*"[Title/Abstract] OR "stroke cerebrovascular"[Title/Abstract] OR "Apoplexy"[Title/Abstract] OR "cerebral stroke*"[Title/Abstract] OR "stroke cerebral"[Title/Abstract] OR "stroke acute"[Title/Abstract] OR "acute stroke*"[Title/Abstract] OR "cerebrovascular accident acute"[Title/Abstract] OR "acute cerebrovascular accident*"[Title/Abstract]  #5 Search: "Cohort Studies"[MeSH Terms] OR "Cross-Sectional Studies"[MeSH Terms] OR "Observational Study"[Publication Type] OR "Observational Studies as Topic"[MeSH Terms] OR "prospectiv*"[Title/Abstract] OR "cohort*"[Title/Abstract] OR "prevalen*"[Title/Abstract] OR "incidenc*"[Title/Abstract] OR "cross-sectional"[Title/Abstract] OR "crosssectional"[Title/Abstract] OR "observation*"[Title/Abstract] OR "longitudinal"[Title/Abstract] OR "epidemiol*"[Title/Abstract]  #6 (#1 OR #2) AND (#3 OR #4) AND #5 |
| Ovid-MEDLINE | # 1exp Sleep Apnea Syndromes/  #2 exp Sleep Apnea, Obstructive/  #3 exp Sleep Apnea, Central/  #4 exp Obesity Hypoventilation Syndrome/  #5 ("Apnea Syndrome, Sleep" or "Sleep Apnea Syndrome" or "Sleep Hypopnea*" or "Hypopnea*, Sleep" or "Apnea*, Sleep" or "Sleep Apnea*" or "Sleep Apnea, Mixed Central and Obstructive" or "Mixed Central and Obstructive Sleep Apnea" or "Sleep Apnea*, Mixed" or "Mixed Sleep Apnea*" or "Hypersomnia with Periodic Respiration" or "Sleep-Disordered Breathing" or "Breathing, Sleep-Disordered" or "Sleep Disordered Breathing" or "Apnea*, Obstructive Sleep" or "Obstructive Sleep Apnea*" or "Sleep Apneas, Obstructive" or "Obstructive Sleep Apnea Syndrome" or "OSAHS" or "Syndrome, Sleep Apnea, Obstructive" or "Sleep Apnea Syndrome, Obstructive" or "Sleep Apnea Hypopnea Syndrome" or "Syndrome, Obstructive Sleep Apnea" or "Upper Airway Resistance Sleep Apnea Syndrome" or "Syndrome, Upper Airway Resistance, Sleep Apnea").ab.  #6 exp Stroke/  #7 exp Cerebrovascular Disorders/  #8 exp Brain Infarction/  #9 exp Brain Ischemia/  #10 exp Ischemic Stroke/  #11 exp Ischemic Attack, Transient/  #12 ("ischemic attack, transient" or "cerebrovascular accident*" or "cva cerebrovascular accident" or "Cerebrovascular Apoplexy" or "apoplexy cerebrovascular" or "vascular accident brain" or "brain vascular accident*" or "cerebrovascular stroke*" or "stroke cerebrovascular" or "Apoplexy" or "cerebral stroke*" or "stroke cerebral" or "stroke acute" or "acute stroke*" or "cerebrovascular accident acute" or "acute cerebrovascular accident*").ab.  #13 ("prospectiv*" or "cohort*" or "prevalen*" or "incidenc*" or "cross-sectional" or "crosssectional" or "observation*" or "longitudinal" or "epidemiol*").ab.  #14 exp Cohort Studies/  #15 exp Cross-Sectional Studies/  #16 exp Observational Study/  #17 exp Observational Studies as Topic/  #18 (1 OR 2 OR 3 OR 4 OR 5) AND (6 OR 7 OR 8 OR 9 OR 10 OR 11 OR 12) AND (13 OR 14 OR 15 OR 16 OR 17) |
| Web of Science | #1 TS=("Sleep Apnea Syndromes" OR "sleep apnea, obstructive" OR "sleep apnea, central" OR"Obesity Hypoventilation Syndrome" OR "apnea syndrome sleep" OR "Sleep Apnea Syndrome" OR "Sleep Hypopnea" OR "hypopnea sleep"OR "hypopneas sleep"OR "Sleep Hypopneas" OR "apnea sleep" OR "apneas sleep"OR "Sleep Apnea" OR "Sleep Apneas" OR "Mixed Central and Obstructive Sleep Apnea" OR "sleep apnea mixed" OR "Mixed Sleep Apnea" OR "Mixed Sleep Apneas" OR "Hypersomnia with Periodic Respiration" OR "sleep disordered breathing" OR "breathing sleep disordered" OR "sleep disordered breathing" OR "apneas obstructive sleep" OR "Obstructive Sleep Apneas"OR "sleep apneas obstructive" OR "Obstructive Sleep Apnea Syndrome"OR "Obstructive Sleep Apnea" OR "OSAHS"OR "syndrome sleep apnea obstructive"OR "sleep apnea syndrome obstructive" OR "apnea obstructive sleep" OR "Sleep Apnea Hypopnea Syndrome" OR "syndrome obstructive sleep apnea"OR "Upper Airway Resistance Sleep Apnea Syndrome")  #2 TS=("Stroke" OR "Cerebrovascular Disorders" OR "Brain Infarction" OR "Brain ischemia" OR "Ischemic Stroke" OR “Hemorrhagic Stroke” OR "ischemic attack, transient" OR "cerebrovascular accident*" OR "cva cerebrovascular accident" OR "Cerebrovascular Apoplexy" OR "apoplexy cerebrovascular" OR "vascular accident brain" OR "brain vascular accident*" OR "cerebrovascular stroke*" OR "stroke cerebrovascular" OR "Apoplexy" OR "cerebral stroke*" OR "stroke cerebral" OR "stroke acute" OR "acute stroke*" OR "cerebrovascular accident acute" OR "acute cerebrovascular accident*")  #3 TS=("Cohort Studies"OR "Cross-Sectional Studies"OR "Observational Study"[Publication Type] OR "Observational Studies as Topic"OR "prospectiv*" OR "cohort*" OR "prevalen*" OR "incidenc*" OR "cross-sectional" OR "crosssectional" OR "observation*" OR "longitudinal" OR "epidemiol*")  #4 #1 AND #2 AND #3 |
| Embase | #1 'sleep apnea syndromes'/exp  #2 'apnea during sleep'/exp OR 'apnea during sleep' OR 'apnea syndrome'/exp OR 'apnea syndrome' OR 'apnea syndromes':ab,ti OR 'apnea, sleep':ab,ti OR 'apneas during sleep':ab,ti OR 'apnoea, sleep':ab,ti OR 'hypopnea during sleep':ab,ti OR 'hypopnea syndrome':ab,ti OR 'hypopneas during sleep':ab,ti OR 'nocturnal apnea':ab,ti OR 'nocturnal apneas':ab,ti OR 'nocturnal apnoea':ab,ti OR 'nocturnal apnoeas':ab,ti OR 'sleep apnea':ab,ti OR 'sleep apnea syndrome':ab,ti OR 'sleep apnea-hypopnea':ab,ti OR 'sleep apnea/hypopnea syndrome':ab,ti OR 'sleep apneas':ab,ti OR 'sleep apnoea':ab,ti OR 'sleep apnoea syndrome':ab,ti OR 'sleep apnoea syndromes':ab,ti OR 'sleep apnoeas':ab,ti OR 'sleep disordered breathing':ab,ti OR 'sleep hypopnea':ab,ti OR 'sleep hypopneas':ab,ti OR 'sleep related breathing abnormalities':ab,ti OR 'sleep related breathing abnormality':ab,ti OR 'sleep related breathing disorder':ab,ti OR 'sleep related breathing disturbance':ab,ti OR 'unspecified sleep apnea':ab,ti OR 'sleep apnea syndromes':ab,ti  #3 'ischemic stroke'/exp OR 'brain hemorrhage'/exp OR 'transient ischemic attack'/exp OR 'brain infarction'/exp OR 'brain ischemia'/exp OR 'cerebrovascular disease'/exp OR 'cerebrovascular accident'/exp  #4'ischemic attack, transient'/exp OR 'ischemic attack, transient' OR 'cerebrovascular accident*':ab,ti OR 'cva cerebrovascular accident':ab,ti OR 'cerebrovascular apoplexy':ab,ti OR 'apoplexy cerebrovascular':ab,ti OR 'vascular accident brain':ab,ti OR 'brain vascular accident*':ab,ti OR 'cerebrovascular stroke*':ab,ti OR 'stroke cerebrovascular':ab,ti OR 'apoplexy':ab,ti OR 'cerebral stroke*':ab,ti OR 'stroke cerebral':ab,ti OR 'stroke acute':ab,ti OR 'acute stroke*':ab,ti OR 'cerebrovascular accident acute':ab,ti OR 'acute cerebrovascular accident*':ab,ti  #5'cohort studies' OR 'cross-sectional studies' OR 'observational study' OR 'observational studies as topic' OR 'prospectiv*' OR 'cohort*' OR 'prevalen*' OR 'incidenc*' OR 'cross-sectional' OR 'crosssectional' OR 'observation*' OR 'longitudinal' OR 'epidemiol*'  (#1 OR #2)AND (#3 OR #4) AND #5 |
| The Cochrane  Library | #1 ('Sleep Apnea Syndromes' OR 'sleep apnea, obstructive' OR 'sleep apnea, central' OR 'Obesity Hypoventilation Syndrome' OR 'apnea syndrome sleep' OR 'Sleep Apnea Syndrome' OR 'Sleep Hypopnea' OR 'hypopnea sleep'OR 'hypopneas sleep'OR 'Sleep Hypopneas' OR 'apnea sleep' OR 'apneas sleep'OR 'Sleep Apnea' OR 'Sleep Apneas' OR 'Mixed Central and Obstructive Sleep Apnea' OR 'sleep apnea mixed' OR 'Mixed Sleep Apnea' OR 'Mixed Sleep Apneas' OR 'Hypersomnia with Periodic Respiration' OR 'sleep disordered breathing' OR 'breathing sleep disordered' OR 'sleep disordered breathing' OR 'apneas obstructive sleep' OR 'Obstructive Sleep Apneas'OR 'sleep apneas obstructive' OR 'Obstructive Sleep Apnea Syndrome'OR 'Obstructive Sleep Apnea' OR 'OSAHS'OR 'syndrome sleep apnea obstructive'OR 'sleep apnea syndrome obstructive' OR 'apnea obstructive sleep' OR 'Sleep Apnea Hypopnea Syndrome' OR 'syndrome obstructive sleep apnea'OR 'Upper Airway Resistance Sleep Apnea Syndrome'):ti,ab,kw 9841  #2 MeSH descriptor: [Ischemic Stroke] explode all trees  #3 MeSH descriptor: [Ischemic Attack, Transient] explode all trees  #4 MeSH descriptor: [Brain Ischemia] explode all trees  #5 MeSH descriptor: [Cerebrovascular Disorders] explode all trees  #6 MeSH descriptor: [Brain Infarction] explode all trees  #7 MeSH descriptor: [Stroke] explode all trees  #8 MeSH descriptor: [Hemorrhagic Stroke] explode all trees  #9 'ischemic attack, transient' OR 'cerebrovascular accident*' OR 'cva cerebrovascular accident' OR 'cerebrovascular apoplexy' OR 'apoplexy cerebrovascular' OR 'vascular accident brain' OR 'brain vascular accident*' OR 'cerebrovascular stroke*' OR 'stroke cerebrovascular' OR 'apoplexy' OR 'cerebral stroke*' OR 'stroke cerebral' OR 'stroke acute' OR 'acute stroke*' OR 'cerebrovascular accident acute' OR 'acute cerebrovascular accident*'  #10 MeSH descriptor: [Cross-Sectional Studies] explode all trees  #11 MeSH descriptor: [Observational Study] explode all trees  #12 'cohort studies' OR 'cross-sectional studies' OR 'observational study' OR 'observational studies as topic' OR 'prospectiv*' OR 'cohort*' OR 'prevalen*' OR 'incidenc*' OR 'cross-sectional' OR 'crosssectional' OR 'observation*' OR 'longitudinal' OR 'epidemiol*'  #13 #1 AND (#2 OR #3 OR #4 OR #5 OR #6 OR #7 OR #8 OR #9) AND (#10 OR #11 OR #12) |
| CINAHL Plus with Full Text | #1 TX 'sleep apnea syndromes' OR TX ( 'Sleep Apnea Syndromes' OR 'sleep apnea, obstructive' OR 'sleep apnea, central' OR'Obesity Hypoventilation Syndrome' OR 'apnea syndrome sleep' OR 'Sleep Apnea Syndrome' OR 'Sleep Hypopnea' OR 'hypopnea sleep'OR 'hypopneas sleep'OR 'Sleep Hypopneas' OR 'apnea sleep' OR 'apneas sleep'OR 'Sleep Apnea' OR 'Sleep Apneas' OR 'Mixed Central and Obstructive Sleep Apnea' OR 'sleep apnea mixed' OR 'Mixed Sleep Apnea' OR 'Mixed Sleep Apneas' OR 'Hypersomnia with Periodic Respiration' OR 'sleep disordered breathing' OR 'breathing sleep disordered' OR 'sleep disordered breathing' OR 'apneas obstructive sleep' OR 'Obstructive Sleep Apneas'OR 'sleep apneas obstructive' OR 'Obstructive Sleep Apnea Syndrome'OR 'Obstructive Sleep Apnea' OR 'OSAHS'OR 'syndrome sleep apnea obstructive'OR 'sleep apnea syndrome obstructive' OR 'apnea obstructive sleep' OR 'Sleep Apnea Hypopnea Syndrome' OR 'syndrome obstructive sleep apnea'OR 'Upper Airway Resistance Sleep Apnea Syndrome' )  #2 TX ischemic stroke OR TX ( brain hemorrhage' OR 'transient ischemic attack' OR 'brain infarction' OR 'brain ischemia' OR 'cerebrovascular disease' OR 'cerebrovascular accident' )  #3 TX 'ischemic attack, transient' OR TX ( 'cerebrovascular accident*' OR 'cva cerebrovascular accident' OR 'cerebrovascular apoplexy' OR 'apoplexy cerebrovascular' OR 'vascular accident brain' OR 'brain vascular accident*' OR 'cerebrovascular stroke*' OR 'stroke cerebrovascular' OR 'apoplexy' OR 'cerebral stroke*' OR 'stroke cerebral' OR 'stroke acute' OR 'acute stroke*' OR 'cerebrovascular accident acute' OR 'acute cerebrovascular accident*' )  #4 TX ( 'Cohort Studies'OR 'Cross-Sectional Studies'OR 'Observational Study'[Publication Type] OR 'Observational Studies as Topic'OR 'prospectiv*' OR 'cohort*' OR 'prevalen*' OR 'incidenc*' OR 'cross-sectional' OR 'crosssectional' ) OR TX ( 'observation*' OR 'longitudinal' OR 'epidemiol*' )  #5 #1 AND (#2 OR #3) AND #4 |

**eTable 2.** Risk of bias in included studies (Joanna-Briggs Institute’s critical appraisal checklist).

| First author | Year | 1 | 2 | 3 | 4 | 5 | 6 | 7 | 8 | 9 |
| --- | --- | --- | --- | --- | --- | --- | --- | --- | --- | --- |
| Zhu et al, 2023 | 2023 | Yes | Yes | Yes | Yes | Yes | Yes | Yes | Yes | No |
| Tayade et al, 2023 | 2023 | Yes | Yes | No | Yes | Unclear | Yes | Yes | Yes | Yes |
| Plomaritis et al, 2023 | 2023 | Yes | Yes | Yes | Yes | Unclear | Unclear | Yes | Yes | Yes |
| Lisabeth et al, 2023 | 2023 | Yes | Yes | Yes | Yes | Unclear | Unclear | Yes | Yes | Yes |
| Bochkarev et al, 2023 | 2023 | Yes | Unclear | Unclear | Yes | No | Yes | Yes | Yes | Yes |
| Patel et al, 2023 | 2023 | Yes | Yes | Yes | Yes | Unclear | Yes | Yes | Yes | No |
| Lin et al, 2023 | 2023 | Yes | Yes | Yes | Yes | Yes | Yes | No | Yes | Unclear |
| Korostovtseva et al, 2023 | 2023 | Yes | Yes | Yes | Yes | Yes | Unclear | Yes | Yes | No |
| Hong et al, 2023 | 2023 | Yes | Yes | Yes | Yes | Unclear | Yes | Unclear | Yes | Unclear |
| Hoang-Anh et al, 2023 | 2023 | Yes | Yes | No | Yes | Unclear | Yes | Yes | Yes | Yes |
| Duss et al, 2023 | 2023 | Yes | Yes | Yes | Yes | Unclear | Yes | Unclear | Yes | Yes |
| Brunetti et al, 2023 | 2023 | Yes | Yes | Yes | Yes | Unclear | Unclear | Yes | Yes | Yes |
| Liu et al, 2023 | 2022 | Yes | Yes | Yes | Yes | Unclear | Yes | Yes | Yes | Yes |
| Zhu et al, 2022 | 2022 | Yes | Yes | Yes | Yes | Yes | Yes | Yes | Yes | Yes |
| Zhang et al, 2022 | 2022 | Yes | Yes | Yes | Yes | Unclear | Yes | Yes | Yes | No |
| Springer et al, 2022 | 2022 | Yes | Yes | Yes | Yes | Unclear | Yes | Yes | Yes | No |
| Simonsen et al, 2022 | 2022 | Yes | Yes | No | Yes | Unclear | Yes | No | Yes | Unclear |
| Schütz et al, 2022 | 2022 | Yes | Yes | Yes | Yes | Unclear | Unclear | Unclear | Yes | No |
| Rafi et al, 2022 | 2022 | Yes | Yes | No | Yes | Unclear | Yes | Unclear | Yes | Yes |
| Huhtakangas et al, 2022 | 2022 | Yes | Yes | Yes | Yes | Unclear | Yes | Yes | Yes | Yes |
| Griesbach et al, 2022 | 2022 | Yes | Yes | No | Unclear | Yes | Yes | Yes | Yes | Yes |
| Edrissi et al, 2022 | 2022 | Yes | Yes | Yes | Yes | Unclear | Yes | Unclear | Yes | No |
| Baillieul et al, 2022 | 2022 | Yes | Yes | Yes | Yes | Yes | Yes | Yes | Yes | Yes |
| Šiarnik et al, 2021 | 2021 | Yes | Yes | Yes | Yes | No | Yes | Yes | Yes | Yes |
| Riglietti et al, 2021 | 2021 | Yes | No | No | Yes | Unclear | Yes | Yes | Yes | Yes |
| Gottlieb et al, 2021 | 2021 | Yes | No | Yes | Yes | No | Unclear | Yes | Yes | Yes |
| Folgueira et al, 2021 | 2021 | Yes | Yes | No | Yes | Yes | Yes | Yes | Yes | Yes |
| Estai et al, 2021 | 2021 | Yes | No | No | Yes | Yes | Yes | Yes | Yes | Yes |
| Domínguez-Mayoral et al, 2021 | 2021 | Yes | Yes | Yes | Yes | Yes | Unclear | Yes | Yes | Unclear |
| Chen et al, 2021 | 2021 | Yes | Yes | Yes | Yes | Yes | Yes | Yes | Yes | Unclear |
| Petrie et al, 2021 | 2021 | No | Yes | No | No | No | Yes | Yes | Yes | Yes |
| Yoon et al, 2020 | 2020 | Yes | Yes | Yes | Unclear | Yes | Yes | Unclear | Yes | Unclear |
| Slim et al, 2020 | 2020 | Yes | Yes | No | Yes | Unclear | Yes | Yes | Yes | Yes |
| Pajediene et al, 2020 | 2020 | Yes | Yes | No | Yes | Yes | Yes | Unclear | Yes | Yes |
| Ott et al, 2020 | 2020 | Yes | Yes | Yes | Yes | No | Unclear | Yes | Yes | Yes |
| McKee et al, 2020 | 2020 | Yes | Yes | Yes | Yes | No | Yes | Yes | Yes | Yes |
| Kisabay Ak et al, 2020 | 2020 | No | Yes | No | Yes | Yes | Yes | Yes | Yes | Yes |
| Huhtakangas et al, 2020 | 2020 | Yes | Yes | Yes | Yes | No | Yes | Yes | Yes | Yes |
| Haula et al, 2020 | 2020 | No | Yes | No | Yes | Yes | Yes | Yes | Yes | Yes |
| Castello-Branco et al, 2020 | 2020 | No | Yes | No | Yes | Yes | Yes | Yes | Yes | Yes |
| Brown et al, 2020 | 2020 | Yes | Yes | Yes | Yes | No | Yes | Yes | Yes | unclear |
| Nair et al, 2019 | 2019 | No | Yes | No | Yes | No | Yes | Yes | Yes | Yes |
| Matsuura et al, 2019 | 2019 | Yes | Yes | Yes | Yes | Yes | Yes | Yes | Yes | Yes |
| Li et al, 2019 | 2019 | No | Yes | No | Yes | No | Yes | Yes | Yes | Yes |
| Brown et al, 2019 | 2019 | Yes | Yes | Yes | Yes | No | Yes | Yes | Yes | Yes |
| Zhang et al, 2018 | 2018 | Yes | Yes | Yes | Yes | No | Yes | Yes | Yes | Yes |
| Yaddanapudi et al, 2018 | 2018 | Yes | Yes | Yes | Yes | No | Yes | Yes | Yes | Yes |
| Tazartukova et al, 2018 | 2018 | No | Yes | No | Unclear | No | Yes | Yes | Yes | Yes |
| Losurdo et al, 2018 | 2018 | Yes | Yes | Yes | Yes | No | Yes | Yes | Yes | Yes |
| Lisabeth et al, 2018 | 2018 | Yes | Yes | Yes | Yes | No | Yes | Yes | Yes | Yes |
| Festic et al, 2018 | 2018 | Yes | Yes | Yes | Yes | No | Yes | Yes | Yes | unclear |
| Slonkova et al, 2017 | 2017 | No | Yes | No | Yes | No | Yes | Yes | Yes | Yes |
| Scherbakov et al, 2017 | 2017 | Yes | Yes | Yes | Yes | No | Yes | Yes | Yes | Yes |
| Sarfo et al, 2017 | 2017 | Yes | Yes | Yes | Yes | Yes | Yes | Yes | Yes | Yes |
| Ryan et al, 2017 | 2017 | No | No | No | Yes | No | Yes | Yes | Unclear | Yes |
| Ponsaing et al, 2017 | 2017 | No | No | No | Yes | Yes | Yes | Yes | Yes | Yes |
| Menon et al, 2017 | 2017 | Yes | No | Yes | Yes | No | Yes | Yes | Yes | Yes |
| Lisabeth et al, 2017 | 2017 | Yes | No | Yes | Yes | No | Yes | Yes | Yes | Unclear |
| Kumar et al, 2017 | 2017 | No | No | No | Yes | Yes | Yes | Yes | Yes | Yes |
| Kim et al, 2017 | 2017 | Yes | Yes | Yes | Yes | No | Yes | Yes | Yes | Yes |
| Huhtakangas et al, 2017 | 2017 | Yes | Yes | Yes | Yes | No | Yes | Yes | Yes | Yes |
| Fisse et al, 2017 | 2017 | Yes | Yes | Yes | Unclear | No | Yes | Yes | Yes | Yes |
| Tur et al, 2016 | 2016 | Yes | Yes | No | Yes | No | Yes | Yes | Yes | Yes |
| Lutohin et al, 2016 | 2016 | No | No | No | Yes | No | Yes | Yes | Yes | Unclear |
| Koo et al, 2016 | 2016 | Yes | Yes | Yes | Yes | No | Unclear | Yes | Yes | Yes |
| Ifergane et al, 2016 | 2016 | No | Yes | No | Yes | Yes | Yes | Yes | Yes | Yes |
| Boulos et al, 2016 | 2016 | No | Yes | No | Yes | No | Yes | Yes | Yes | Yes |
| Stahl et al, 2015 | 2015 | No | Yes | Yes | Yes | Yes | Yes | Yes | Yes | Yes |
| Chen et al, 2015 | 2015 | Yes | Yes | Yes | Yes | No | Yes | Yes | Yes | Yes |
| Väyrynen et al, 2014 | 2014 | No | Yes | No | No | Yes | Yes | Yes | Unclear | Yes |
| Shibazaki et al, 2014 | 2014 | Yes | Yes | No | Yes | No | Yes | Yes | Yes | Yes |
| Ramos et al, 2014 | 2014 | Yes | Yes | Yes | Yes | No | Yes | Yes | Yes | Yes |
| Lefèvre-Dognin et al, 2014 | 2014 | No | Yes | No | Yes | No | Yes | Yes | Yes | Yes |
| Kepplinger et al, 2014 | 2014 | Unclear | Yes | Yes | Yes | No | Yes | Yes | Yes | Yes |
| Shibazaki et al, 2014 | 2013 | Yes | Yes | Yes | No | Yes | Yes | Yes | Yes | Yes |
| Ciccone et al, 2014 | 2013 | Yes | Yes | Yes | Yes | No | Yes | Yes | Yes | Yes |
| Cereda et al, 2014 | 2013 | No | Yes | No | Yes | No | Yes | Yes | Yes | Unclear |
| Ahn et al, 2014 | 2013 | Yes | Yes | Yes | Yes | No | Yes | Yes | Yes | Yes |
| Xu et al, 2014 | 2012 | No | Yes | No | Yes | No | Yes | Yes | Yes | Yes |
| Hsieh et al, 2014 | 2012 | Yes | Yes | Yes | Yes | No | Yes | Yes | Yes | Yes |
| Camilo et al, 2014 | 2012 | No | Yes | Yes | Yes | No | Yes | Yes | Yes | Yes |
| Aaronson et al, 2014 | 2012 | No | Yes | No | Yes | No | Yes | Yes | Yes | Yes |
| Chen et al, 2014 | 2011 | Yes | Yes | Yes | No | Yes | Yes | Yes | Yes | Yes |
| Chan et al, 2014 | 2010 | No | Yes | No | Yes | No | Unclear | Yes | Yes | Yes |
| Brooks et al, 2014 | 2010 | No | Yes | No | Yes | No | Yes | Yes | Yes | Yes |

#Critical appraisal checklist (Munn et al., 2015)/1.Was the sample frame appropriate to address the target population? /2.Were the study participants sampled in an appropriate way? /3. Was the sample size adequate? /4. Were the study subjects and the setting described in detail?/5.Was the data analysis conducted with sufficient coverage of the identified sample? /6.Were valid methods used for the identification of the condition? /7.Was the condition measured in a standard, reliable way for all participants? /8.Was there appropriate statistical analysis? /9.Was the response rate adequate, and if not, was the low response rate managed appropriately? /Prespecified domains for quality assessment: a) participants (questions 1, 2, 4, and 9); b) outcome measurement (questions 6 and 7); and c) statistics (questions 3, 5, and 8).

**eTable 3.** Results of multivariate meta-regression analysis of SDB.

|  | B | 95%CI | t | *P* | Adjusted R^2^ |
| --- | --- | --- | --- | --- | --- |
| Gender(male%) | 1.54 | 1.43, 1.67 | 11.30 | ＜0.0001 | 64.31% |
| Sample size | 0.99 | 0.99,1.01 | -0.24 | 0.01 | 10.24% |
| Publication year | 0.16 | -0.054, 0.851 | 0.45 | 0.657 |  |
| SDB assessment | -0.086 | -0.191, 0.19 | -1.63 | 0.108 |  |
| Phases of stroke | -0.0098 | -0.069, 0.49 | -0.33 | 0.742 |  |
| Region | -0.029 | -0.085, 0.026 | -1.06 | 0.292 |  |

|  |
| --- |
| eFigure 1. Forest plot of prevalence of mild SDB and moderate-severe SDB among stroke patients |
